# The role of antenatal corticosteroids in twin pregnancy

**DOI:** 10.3389/fphar.2023.1072578

**Published:** 2023-02-02

**Authors:** Jie Zhu, Shuyue Li, Ying Zhao, Yu Xiong

**Affiliations:** ^1^ Obstetrics and Gynecology Hospital, Fudan University, Shanghai, China; ^2^ The Shanghai Key Laboratory of Female Reproductive Endocrine-Related Diseases, Shanghai, China

**Keywords:** antenatal corticosteroids, twin pregnancies, respiratory distress syndrome, corticosteroids, preterm birth

## Abstract

Twin pregnancy was associated with significantly higher rates of adverse neonatal and perinatal outcomes. One of the underlying causes is that twins are prone to preterm birth. Antenatal corticosteroids are widely used for reducing the incidence of neonatal respiratory distress syndrome initially and other neonatal mortality and morbidities subsequently. As it is widely used as a prophylactic treatment for potential premature births, there remain controversies of issues relating to twin gestations, including window for opportunity, timing of use, repeat course, optimal administration-to-delivery intervals, dosage, and type of corticosteroid. Thus, we present a thorough review of antenatal corticosteroids usage in twin gestation, emphasizing the aforementioned issues and attempting to offer direction for future investigation and clinical practice.

## 1 Introduction

Antenatal corticosteroids (ACS) are used for accelerating fetal lung maturation for women at risk of premature births (Effect of Corticosteroids for Fetal Maturation on Perinatal Outcomes, 1995). The initial indication for ACS was reducing the incidence of neonatal respiratory distress syndrome (RDS) (Roberts and Dalziel, 2006), which is a serious complication of preterm birth (PTB) and the primary cause of early neonatal death and disability. And ACS was subsequently extended to generally reduce neonatal mortality and morbidities like serious neurological and abdominal problems including the risk of intraventricular hemorrhage (IVH) and necrotizing enterocolitis (NEC) (Roberts et al., 2017). A course of ACS includes two doses of betamethasone acetate/phosphate 12 mg IM 24 h apart, or two doses of dexamethasone phosphate 12 mg IM 24 h apart and is recommended for women with a high likelihood of preterm birth from 24 to 34 weeks of gestation irrespective of whether single or multiple birth is anticipated by authorities (WHO, 2022; Norman et al., 2021; Stock et al., 2022; American College of and Gynecologists' Committee on Practice Bulletins-Obstetrics SfM-, 2021).

The rates of PTB (28–36 weeks of gestation, 37.1%) and early PTB (28–33 weeks of gestation, 13.0%) among twin pregnancies were substantially higher than those among singletons (7.3% for PTB and 2.4% for early PTB) according to WHO Multicountry Survey on Maternal and Newborn Health (Santana et al., 2018). As twins tend to have an earlier gestational age of delivery, mortality and morbidity following premature birth can carry enormous consequences for societies and economies. Thus, ACS, as one of the tertiary prevention interventions, should be properly provided to overcome the immediate and future health challenges of premature twin newborns.

**FIGURE 1:**
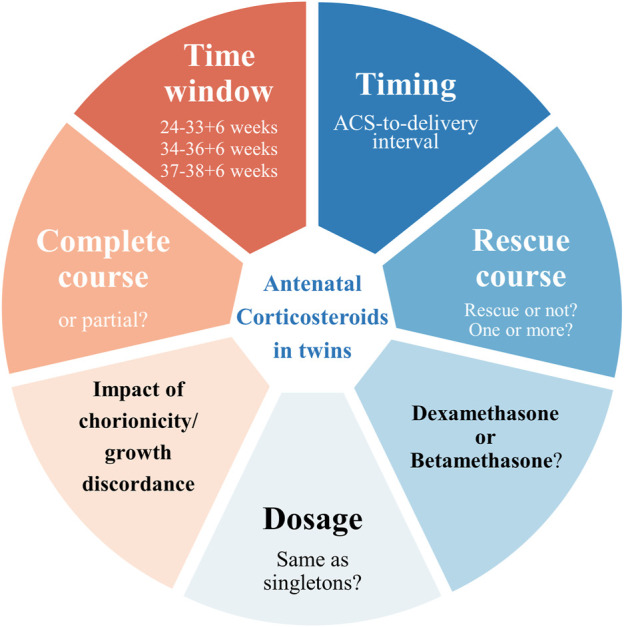
Controversies of antenatal corticosteroids in twin pregnancy.

While the efficacy of ACS treatment in singletons is supported by abundant evidence, the current proof of which in twin gestations is relatively limited and less consistent. There remain controversies in various aspects regarding twin gestations, including but not limited to window for opportunity, timing of use, repeat course, optimal ACS-to-delivery intervals, dosage and type of corticosteroid (Figure 1). We here present a comprehensive review of ACS in twin gestation based on the evidence when possible, emphasizing the controversies above and their influence on the efficacy and safety of ACS, and aimed to provide direction for subsequent investigations and recommendations for clinicians.

## 2 Effects of ACS

### 2.1 Therapeutic effects of ACS

Corticosteroids are easily transported across the placenta. They act to promote the production and secretion of alveolar surfactant, decrease vascular permeability, and increase compliance and maximal lung volume of fetal lungs according to human and animal studies (Ballard and Ballard, 1995), which can prevent respiratory-related morbidity and mortality affecting preterm newborns. Meanwhile, the increased corticosteroids support other physiologic changes that facilitate neonatal adaption, especially the very preterm infants, including energy substrate metabolism control, increased blood pressure, and enhanced cardiovascular adaptation (Hillman et al., 2012).

In twins, evidence from a meta-analysis of non-randomized studies published in 2022 suggests that ACS are associated with a lower incidence of neonatal mortality and RDS, which was in agreement with the 2020 Cochrane meta-analyses of randomized controlled trials (RCTs) in multiples, but was not convincingly associated with other outcomes including IVH, NEC, bronchopulmonary dysplasia (BPD), periventricular leukomalacia (PVL), and retinopathy of prematurity (ROP) (McGoldrick et al., 2020; Socha et al., 2022) probably due to the paucity of studies focusing on these secondary outcomes in twin pregnancy (Table 1).

**TABLE 1:** Overall odds of outcomes among twin neonates who were exposed to antenatal corticosteroids (ACS) vs. those unexposed to ACS.

Outcomes	aOR (95% CI)	I^2^ (%)	Study number	Neonate number
Neonatal mortality	0.59 (0.43–0.80)	69	5	20,312
RDS	0.70 (0.57–0.86)	67	7	20,628
IVH	0.78 (0.55–1.13)	80	3	12,497
NEC	1.02 (0.83–1.25)	0	4	19,773
BPD	1.08 (0.96–1.21)	0	5	19,106
PVL	0.77 (0.57–1.03)	56	3	11,411
ROP	0.96 (0.84–1.09)	0	4	18,514

Data abstracted from Socha et al. (2022).

aOR, Adjusted odds ratios; RDS, Respiratory distress syndrome; IVH, Intraventricular hemorrhage; NEC, Necrotizing enterocolitis; BPD, Bronchopulmonary dysplasia; PVL, Periventricular leukomalacia; ROP, Retinopathy of prematurity.

### 2.2 Potential adverse effects of ACS

Epidemiological evidence and animal work indicate a possible link between ACS exposure and potential harm.

#### 2.2.1 Short-term risks

Glucocorticoids are recognized to have an adverse effect on growth in animal studies (Braun et al., 2007). In humans, in line with the previous studies (Murphy et al., 2008; Braun et al., 2015), a recent observative study noted that ACS was consistently associated with a reduction in birth size for infants born preterm, near term, or at term (Rodriguez et al., 2019). And it has been suggested that there is a significant reduction in fetal growth velocity including head circumference, abdominal circumference, and estimated fetal weight in pregnancies exposed to ACS (Rizzo et al., 2022). As for twin pregnancies, ACS was found to reduce birth size of preterm neonates in a dose-dependent manner (Braun et al., 2016). Therefore, it is worth attention as impaired growth during pregnancy and infancy is in turn closely associated with neurodevelopmental disorders in the future.

In several studies including a large multi-center randomized controlled trial, hypoglycemia was noted in late preterm infants whose mothers were treated with ACS (Kamath-Rayne et al., 2012; Gyamfi-Bannerman et al., 2016; Ramadan et al., 2016). The finding was the same in twins as two retrospective cohort studies showed (Ben-David et al., 2020; Vieira et al., 2022). Despite appearing self-limiting, hypoglycemia has been connected to poor neurological outcomes in premature newborns (McKinlay et al., 2017), to which thus attention should be paid.

#### 2.2.2 Long-term risks

Negative programming signals may cause long-lasting maladaptive alterations, raising the risk of physical, mental, and developmental impairments in offspring, which has been convinced by numerous animal studies (Quinlivan et al., 2000; Church et al., 2012; Carson et al., 2016). Human evidence is still limited, but it is gradually accumulating and leading to some warning signals. Recent follow-up studies and retrospective cohort studies have shown that exposure to ACS during pregnancy was significantly associated with mental and behavioral disorders from childhood to adolescence (Alexander et al., 2012; Asztalos et al., 2013; Stutchfield et al., 2013; Ilg et al., 2019; Melamed et al., 2019; Raikkonen et al., 2020; Wolford et al., 2020). Although several studies above included twins, no one provided disaggregated estimates in twins, which allows for a great deal of exploration potential.

In animal studies, ACS exposure may result in changes in several aspects like vascular function (Pulgar and Figueroa, 2006), elastin synthesis (Swee et al., 1995), and renin-angiotensin system (Yu et al., 2018), which finally cause long-term cardiovascular disorders. In humans, higher systolic and diastolic blood pressures (Doyle et al., 2000), decreased heart rate variability (Nixon et al., 2017) and changed renin angiotensin aldosterone system (South et al., 2017) were found in 14-year-old children who were exposed to ACS, despite few children having overt hypertension. Decreased aortic distensibility, which is a risk factor for cardiovascular diseases, was also found among ACS-exposed young adults (Kelly et al., 2012).

## 3 The choice of corticosteroid

Though ACS is frequently used to prevent adverse outcomes in preterm newborns, there is still no agreement on the type of corticosteroid to use. Opinion leaders, local accessibility, and cost are some of the variables influencing the decision. Dexamethasone and betamethasone have been used most widely worldwide. The 2022 Cochrane meta-analyses which included 11 trials found no evidence of a difference between dexamethasone and betamethasone for infant outcomes including RDS, IVH, chronic lung disease, and maternal outcomes like chorioamnionitis (Williams et al., 2022). And as for the long-term child outcomes, there was only a multi-center randomized controlled trial which came up with the conclusion that the incidence of survival without neurosensory disability at age 2 years did not differ between dexamethasone and betamethasone treatment (Crowther et al., 2019a). There were no trials that reported the effects of these medicines beyond 2 years of age.

Despite the fact that there was no difference between these drugs for the majority of maternal, neonatal, and early childhood outcomes, the evidence was ambiguous and left open the possibility of significant benefits or harms for a number of vital outcomes for the mother, infant, child, and adult. Overall, it remains unclear whether there are important differences between dexamethasone and betamethasone.

## 4 ACS administration patterns in twin pregnancies

Since clinical efficacy and safety of ACS above are closely related to its pattern of use including time window, course of treatment, optimal ACS-to-delivery interval, and dosage, these aspects need to describe in detail.

### 4.1 Time window

#### 4.1.1 From 24 + 0 to 33 + 6 weeks of gestation

A single course of corticosteroids is recommended for pregnant women between 24 0/7 and 33 6/7 weeks of gestation who are at risk of preterm delivery within 7 days, regardless of fetal number (Committee on Obstetric P. Committee Opinion No, 2017). Although the evidence supporting this recommendation came from RCTs, select observational studies, and extrapolation from studies on singletons, subsequent research on twin pregnancies has also shown that ACS in twins can have the same or comparable efficacy for RDS and neonatal mortality as in singletons (Hacking et al., 2001; Blickstein et al., 2005; Kuk et al., 2013; Melamed et al., 2016; Riskin-Mashiah et al., 2018; Vaz et al., 2018; Kong et al., 2020; Mwita et al., 2022).

#### 4.1.2 Late preterm birth (from 34 + 0 to 36 + 6 weeks of gestation)

After the results of the Antenatal Late Preterm Steroid (ALPS) study published, which advocated the administration of betamethasone to women at risk for late preterm delivery significantly reduced the rate of neonatal respiratory complications in singletons (Gyamfi-Bannerman et al., 2016), change happened in clinical practice, with the American College of Obstetricians and Gynecologists (ACOG) releasing a committee opinion in 2017 recommending treating women at risk of imminent birth in the late preterm period, who had not received prior ACS, with a course of ACS(44). Since then, there has been a continual stream of research on the efficacy and the short-term and long-term adverse effects of ACS in late preterm birth, emphasizing hypoglycemia and adverse neurodevelopmental outcomes (Saccone and Berghella, 2016; Deshmukh and Patole, 2021; Sarid et al., 2022). However, overwhelming emphasis has been placed on singletons. Whether these findings also apply to late-preterm twins is currently unclear. For twins, few studies are focusing on those who received ACS after 34 + 0 weeks of gestation and delivered during the late preterm period. A retrospective cohort study including a total of 290 women with twin pregnancies showed that late preterm ACS administration in twins complicated by late preterm birth did not reduce neonatal respiratory morbidity but was associated with higher rates of neonatal intensive care unit (NICU) admission and hypoglycemia (Ben-David et al., 2020). Another study including 1,032 mother-child pairs in twins showed that exposure to ACS is associated with increased odds of hypoglycemia, but no significant association with respiratory complications (Vieira et al., 2022). So far, the results of ACS for late preterm birth of twins have not been very promising. And there is a study called ACTWIN (Antenatal Corticosteroids in TWIN late preterm neonates) trial which might be the first randomized controlled trial that evaluates the effectiveness of ACS in late-preterm twin neonates (Hong et al., 2019).

#### 4.1.3 Early term birth (from 37 + 0 to 38 + 6 weeks of gestation)

Infants born in the early term period are more likely to suffer respiratory morbidity, particularly if born *via* caesarean section prior to the onset of labor (Zanardo et al., 2004). Hence, it is meaningful to determine whether ACS could benefit this population with respiratory complications. To date, limited evidence in singletons available suggests that ACS before elective caesarean section at early term does not reduce rates of RDS (Stutchfield et al., 2005; Ahmed et al., 2015), but was associated with a reduction in school performance (Stutchfield et al., 2013). However, no completed or ongoing published studies focusing on ACS for elective caesarean section at early term among twins have been found as yet.

In summary, ACS is recommended for twin pregnancies with a high likelihood of preterm birth from 24 to 34 weeks of gestation based on the current evidence. For those at risk of late preterm birth, ACS administration remains debatable. And there is no reason for advocating ACS at this time when at risk of early term delivery.

### 4.2 ACS-to-delivery interval

Another contributing factor to unpredictability is the ACS-to-delivery interval. So far, it is advised by guidelines and consensus that the corticosteroids should be given within 7 days before preterm birth (Committee on Obstetric P. Committee Opinion No, 2017; Medicine FWGoGCPiM-F, 2019; Stock et al., 2022). However, the recommendation mentioned above is based on studies in singletons. Recently, a couple of studies based on twins have emerged for the optimal timing of ACS. In twin pregnancies, ACS treatment was associated with a decreased rate of RDS and in-hospital mortality when the ACS-to-delivery interval was ≤7 days (Melamed et al., 2016; Palas et al., 2018; Vaz et al., 2018), even only in the interval between 2 and 7 days (Kuk et al., 2013). In another research, infants in the ACS-to-delivery interval ≥7 days group even suffered from respiratory disorders significantly more often and were hospitalized longer (Kosinska-Kaczynska et al., 2016).

To sum up, it seems that ACS might be most beneficial when administered within 7 days before birth in twins, which is similar to singletons. However, all the studies of twins currently were observational, contained small sample sizes, and thus had weak evidence. It is critical to determine the optimal ACS-to-delivery interval for the reason that the ACS response interval is important for considerations of the timing of deliveries and repeat ACS treatments. As twin pregnancy is an independent risk factor for suboptimal ACS administration (Rottenstreich et al., 2018), targeted measures should be implemented to promote the utilization of ACS in this susceptible population of women.

### 4.3 Complete course

In 2003, an incomplete course of ACS was proven to be beneficial to perinatal morbidity and mortality (Elimian et al., 2003). Regarding twin births, the results were somewhat different. Blickstein et al. (2005) found that a complete course of ACS significantly reduced the incidence of RDS in twins, whereas partial treatment had the same effect as no treatment. Another study that also considered the gestational age at delivery showed that a complete course of ACS was associated with a reduction in death and RDS in multiple infants born at 28–34 weeks’ gestation, but the incomplete course was not (Herrera et al., 2019). Nevertheless, the administration of even a single dose of ACS to a mother in danger of imminent delivery before 34 weeks of gestation should be strongly considered by clinicians as large sample studies comparing the efficacy of complete and incomplete ACS in twins are still lacking. Where it is deemed safe, tocolytic therapy should be taken into consideration as an intervention to gain time to complete a single course of ACS (WHO, 2022).

### 4.4 Rescue course

Due to worries about maternal and fetal impairment and the imbalance of benefits and risks, planned multiple courses are not advised. However, given the difficulty to predict preterm birth in twins, a rescue course of ACS is worth considering.

An individual participant data meta-analysis of RCTs including 5,915 infants demonstrated that repeat prenatal corticosteroids given to women at ongoing risk of preterm birth after an initial course reduce the need for respiratory support, although birth weight z-scores were reduced (Crowther et al., 2019b). And there were no significant differences in treatment effects among the subgroups by the number of fetuses *in utero*. As for twins, a rescue ACS course was associated with a lower rate of RDS, surfactant use, and BPD, while no differences were noted in neonatal birthweight, head circumference, and the rate of neonatal hypoglycemia (Zigron et al., 2022), which was supported by another retrospective study (Bibbo et al., 2013). But the results of these studies above may be limited by retrospective design and small sample size. Although further studies are warranted to determine the effect of rescue corticosteroids in twin pregnancies, in women who are less than 34 weeks of gestation, at risk of preterm delivery within 7 days, and whose previous course of ACS was administered more than 14 days ago, a single repeat course of ACS should be taken into consideration.

### 4.5 Impact of chorionicity

The effect of chorionicity has not been widely appreciated in population studies and no association was found between chorionicity and the efficacy and ACS in population-based studies. Yet, data from a pharmacokinetic study suggest that the presence of two foetoplacental units in dichorionic (DC) twin pregnancies may increase the betamethasone metabolism by hepatic CYP3A4 and/or placental 11β-HSD2 enzymes (Rodrigues et al., 2022). Therefore, further pharmacological studies are needed to investigate whether these betamethasone pharmacokinetic changes require dose adjustment in DC twin pregnancies. And clinical studies with higher levels of evidence are urgently needed.

### 4.6 Impact of growth discordance

Growth discordance, another characteristic of twin pregnancy, makes it more challenging to determine the exact gestational age. The question of whether the crown-rump length of the larger or smaller fetus should be used as a standard for establishing gestational age is still up for debate (Salomon et al., 2005; Khalil et al., 2016; DeYoung et al., 2021). To guarantee that all eligible women receive ACS while preventing unnecessary treatment of ineligible women, accurate and standardized gestational age assessment is vital. However, studies examining the impact of ACS in groups with growth discordance are scarce. This would be an indication of ACS in twins if it improves the outcomes for smaller fetuses without raising the danger for larger fetuses. To determine this, however, large sample studies are required.

### 4.7 Dosage

The optimal corticosteroid dose to use remains unclear. The International Federation of Gynecology and Obstetrics recently recommended the common regimens of two doses of betamethasone 12 mg given intramuscularly 24 h apart and two doses of dexamethasone 12 mg given intramuscularly 24 h apart, although four doses of dexamethasone 6 mg given intramuscularly 12 h apart has also been recommended. There aren’t any differences in the guidelines for administering ACS between multiple pregnancies and singletons, even for the dosage of ACS. A clinical trial from the view of pharmacokinetics published in 2002 pointed out that the shorter half-life of betamethasone in twin pregnancy than in singleton pregnancy may cause the level of betamethasone to be subtherapeutic for lung maturation in twin pregnancy (Ballabh et al., 2002). But in later studies, maternal and umbilical cord blood serum betamethasone concentrations and pharmacokinetics were the same as singletons in twin pregnancies (Della Torre et al., 2010). Thus, if there is no conclusive difference between singletons in twin pregnancies, then the ACS administration of twins may need to continue to follow the guidelines for singletons.

## 5 Summary

The clinical practice of ACS in twins is almost an extrapolation of that in singletons, but a translation like that could not always be suitable. In the overall population, twin pregnancies are relatively uncommon, thus they are frequently undervalued in clinical studies. Meanwhile, plenty of clinical research only considers singleton pregnancies and excludes twin pregnancies due to the biological differences between them. Nonetheless, future research has the potential to full the gaps in the administration of ACS in twins: whether or not to use it, the actual impact of possible contributing factors, and the long-term outcomes. Firstly, as new trials for fetuses <34 weeks’ gestation were viewed as unethical, it is still reasonable for trials to assess the effect of ACS in late-preterm (34 + 0 to 36 + 6 weeks of gestation) and early-term twins (37 + 0 to 38 + 6 weeks of gestation). Secondly, as timing, the number of courses, dosage, and chorionicity likely affect the association between ACS and outcomes of interest, research containing sufficiently granular data is needed. Thirdly, the follow-up studies spanning childhood, adolescence, and adulthood are required to determine the long-term effects of ACS. We believe that this review is essential for a thorough grasp of the current situation and to determine a direction for future clinical practice and research in ACS of twin pregnancies.
